# Supplementary material for: Signal integration and information transfer in an allosterically regulated network
Source: NPJ Syst Biol Appl. 2019 Jul 18;5:23. doi: 10.1038/s41540-019-0100-9 (PMC6639376; doi:10.1038/s41540-019-0100-9)
Supplement: Supplementary file 1 — Supplementary material [file 41540_2019_100_MOESM1_ESM.pdf]

Signal integration and information transfer in an allosterically regulated network (Supplement)

Erin M. Shockley, Carol A. Rouzer, Lawrence J. Marnett, Eric J. Deeds, and Carlos F. Lopez

June 10, 2019

Supplementary Notes

Model Creation and Calibration

The COX-2 reaction model (CORM) was implemented in PySB [2]. When executed, CORM generates a graph of biochemical reactions that is in turn converted into 13 ordinary differential equations, using the mass-action kinetics formalism. Model calibration to experimental data was performed using the PyDREAM package [4], available at `http://github.com/LoLab-VU/PyDREAM`. Experimentally measured parameters were fixed. Fitted parameters included both K_D and k_{cat} values. Because the ordinary differential equations used to derive CORM require rate constants instead of K_D values, fitted K_D values were converted into rate parameters (k_r/k_f). All reaction k_fs were assumed to be diffusion limited, and the k_r was varied to yield a particular K_D. In addition to experimental data constraints, simulations were also constrained thermodynamically using the presence of four cycles within the network of interactions. The final ensemble of parameter values obtained after model calibration is available at `http://github.com/LoLab-VU/CORM/raw/master/results/2015_02_02_COX2_all_traces.npy`. and in the supplementary data. In addition, the credible intervals for each parameter value determined during parameter calibration are indicated in Table S1. The steps detailed in this section are parts (1) and (2) of Fig. S3.

Parameter	Lower Bound	Upper Bound
kcat_AA2	0.92	17
kcat_AA3	1.3	1.7
kcat_AG3	0.14	0.73
KD_AA_allo1	4.9	3700
KD_AA_allo2	1.2	2.9
KD_AA_allo3	0.20	1.2
KD_AA_cat2	0.0	0.060
KD_AA_cat3	0.0	0.24
KD_AG_allo1	13	5400
KD_AG_allo2	0.23	4.6
KD_AG_cat2	0.010	3.9
KD_AG_cat3	0.0	0.070

Table S1: Credible Intervals for Parameters in CORM

Model Simulations

All CORM simulations were run using the PySB [2] SciPy-based integrator. PySB version 1.1 (available at `http://github.com/LoLab-VU/pysb`) and CORM version 1.0 (available at `http://github.com/LoLab-VU/CORM`) were used. A detailed description of the CORM is available in the supplementary material in [4] For flux analysis, species and reaction trajectories were generated at twelve time points between .01 and ten seconds. Calibrated parameter values from previous work [3] were used; Credible intervals for the ensemble of parameter values (taken from [3]) are shown in Table S1. Catalytic rates are in units of s^{-1} and disassociation constants in units of μM.

Pathway Analysis

If one considers the network of biochemical interactions included in CORM, there are a multitude of paths from enzyme and substrates to either possible product. To enumerate these paths, we employ a graph-theory based approach. In this approach, a node-edge graph is constructed in which each node is a set of reactants or products, and each edge represents a reaction connecting a reactant-product set. The edges are directed with the direction dictated by the net integrated reaction flux ($k_f - k_r$) at a given time; these edges are therefore time dependent given the time dependence of the net

reaction flux. We then determine all simple paths (paths with no repeating reactant-product nodes) connecting initial reactants (COX-2 + AA or COX-2 + 2-AG in this case) to final products (PG and PGG) via the net integrated flux directed edges. The relationship between paths then allow us to calculate the proportion of flux each path contributes to the final product as the joint probability of all reactions that constitute that path (see Fig. S4*B*). For example, in Fig. S4*B*, the pathway marked in blue flows from reactants to products through the center node of the network. The first node in this pathway (after reactants) has only one edge leading into it; therefore the probability of selecting that path into that node is 1. The second node has two edges leading into it, one with probability .66 and one with probability .33). The blue path uses the former rather than the latter edge. The final node in the blue path (the products node) can be reached from one of three edges; in this example each are equally probable. Therefore, to calculate the overall probability of selecting the blue pathway through the network, one multiplies the probabilities of selecting each path through the nodes ($\sim 1 \times .66 \times .33$ in the case of the blue path). This analysis is part (3a) of Fig. S3.

Channel Capacity Calculations

Channel capacities were estimated using the EstCC package introduced in [5], which calculates mutual information between a channel input and output using the method of [1]. EstCC input data was comprised of the results of CORM simulations at a given calibrated parameter vector for 500 different initial AA values drawn from μM $U(0, 16)$, after a ten second simulation. For independent input values, 2-AG values were also drawn from μM $U(0, 16)$. For strongly correlated inputs, 2-AG values were set to the value of AA. AA or 2-AG was considered the signal and the concentration of intermediate or final PG/PGG product the response. The number of signal bins and the number of response bins was selected with EstCC using the procedure described in [5]. The estimate of channel capacity for a given input (and input correlation) and output was performed separately for simulations with 5000 different calibrated parameter sets to allow estimation of the uncertainty in channel capacity stemming from parameter uncertainty.

For the channel capacity calculations in a given region of substrate space, calculations were performed identically to the original analysis, except that input values were constrained to low (0-8 μM) or high (8-16 μM) levels of

AA and 2-AG, and only 500 calibrated parameter sets were used to estimate uncertainty from parameter uncertainty (to make the calculations computationally feasible).

In total all channel capacity calculations required approximately 1.5 million CPU hours to complete. Computation was performed using a dynamically scaled Amazon Web Services (AWS) Batch cluster containing up to 20,000 CPUs. This analysis is step (3b) in Fig. S3.

An overview of the entire process of modeling, calibration, and analysis, with steps as referenced in the above text, is shown in Figure S3.

References

[1] Raymond Cheong, Alex Rhee, Chiaochun Joanne Wang, Ilya Nemenman, and Andre Levchenko. Information transduction capacity of noisy biochemical signaling networks. *Science*, 334(6054):354–358, October 2011.

[2] Carlos F Lopez, Jeremy L Muhlich, John A Bachman, and Peter K Sorger. Programming biological models in Python using PySB. *Molecular Systems Biology*, 9(1):646–646, January 2013.

[3] Michelle M Mitchener, Daniel J Hermanson, Erin M Shockley, H Alex Brown, Craig W Lindsley, Jeff Reese, Carol A Rouzer, Carlos F Lopez, and Lawrence J Marnett. Competition and allostery govern substrate selectivity of cyclooxygenase-2. *Proceedings of the National Academy of Sciences of the United States of America*, 112(40):12366–12371, October 2015.

[4] Erin M Shockley, Jasper A Vrugt, and Carlos F Lopez. PyDREAM: high-dimensional parameter inference for biological models in python. *Bioinformatics*, 18:343, 2017.

[5] Ryan Suderman, John A Bachman, Adam Smith, Peter K Sorger, and Eric J Deeds. Fundamental trade-offs between information flow in single cells and cellular populations. *Proceedings of the National Academy of Sciences of the United States of America*, 114(22):5755–5760, May 2017.

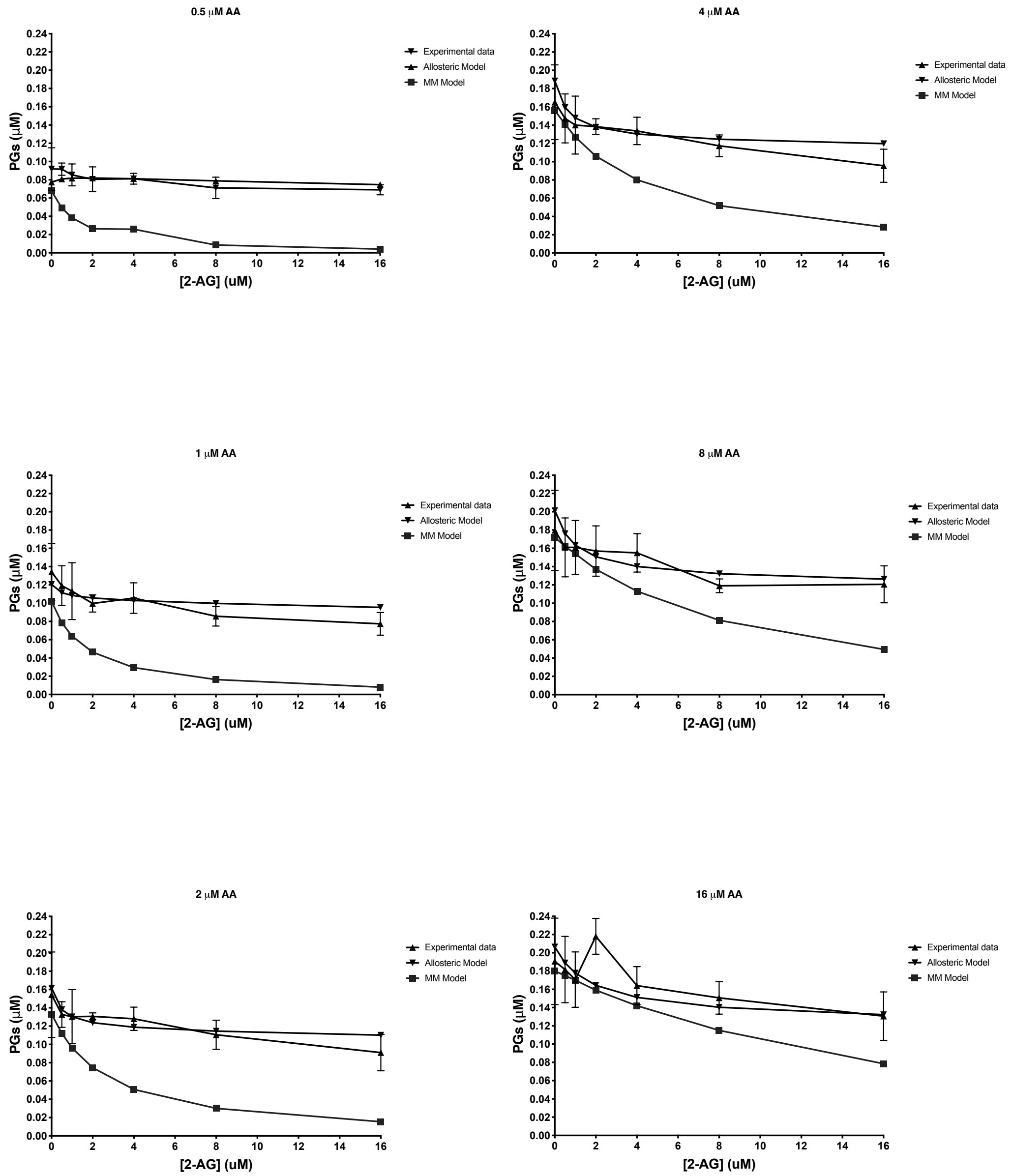

Figure S1: PG Experimental Data and Model-based Simulations. Experimental data and simulations of PG levels in allosteric or Michaelis-Menten based competitive model. Figure adapted from reference: Mitchener, M. M. *et al.* Competition and allostery govern substrate selectivity of cyclooxygenase-2. *Proc. Natl. Acad. Sci. U.S.A.* 112, 12366–12371 (2015).

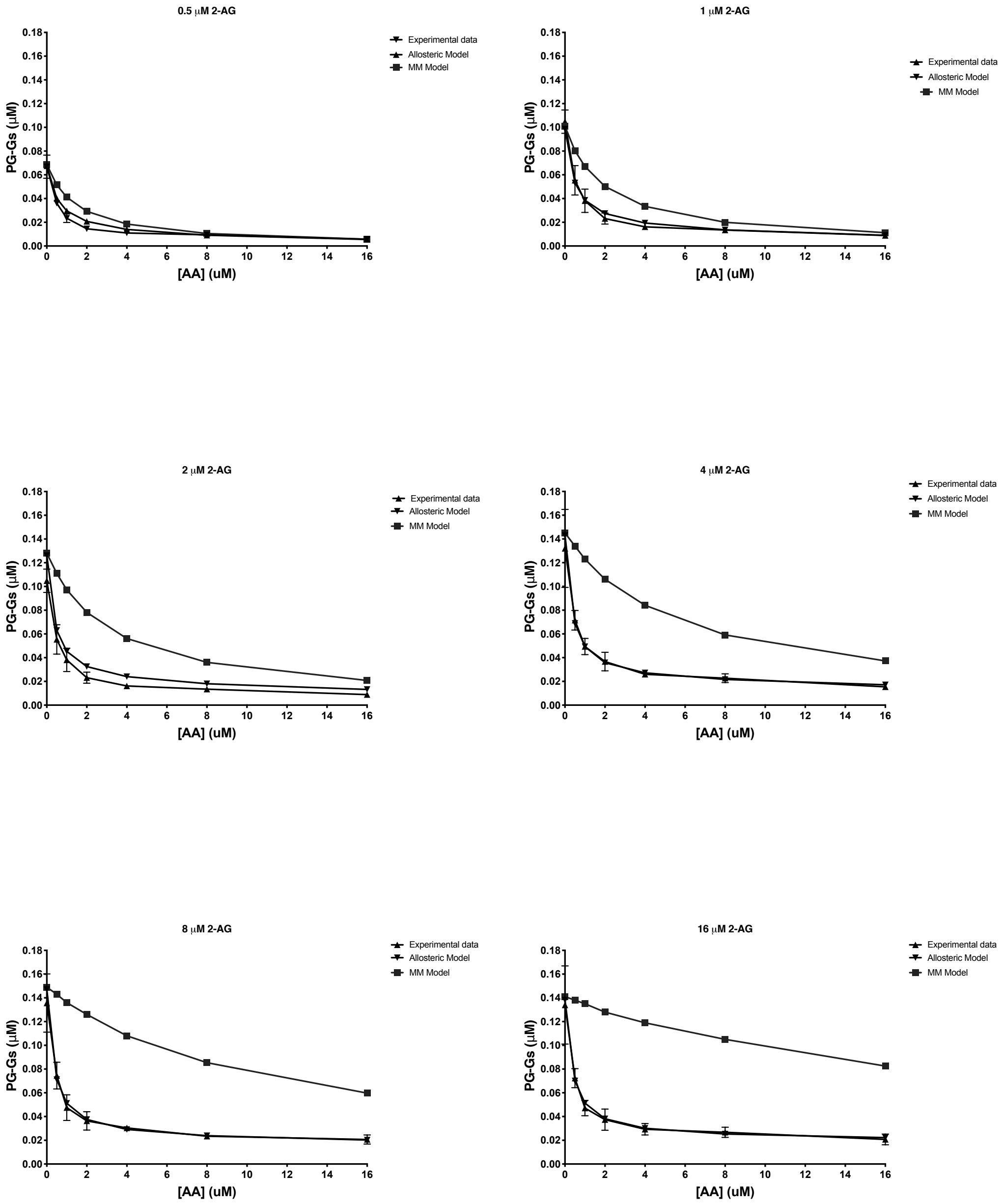

Figure S2: PGG Experimental Data and Model-based Simulations. Experimental data and simulations of PGG levels in allosteric or Michaelis-Menten based competitive model. Figure adapted from reference Mitchener, M. M. *et al.* Competition and allostery govern substrate selectivity of cyclooxygenase-2. *Proc. Natl. Acad. Sci. U.S.A.* 112, 12366–12371 (2015).

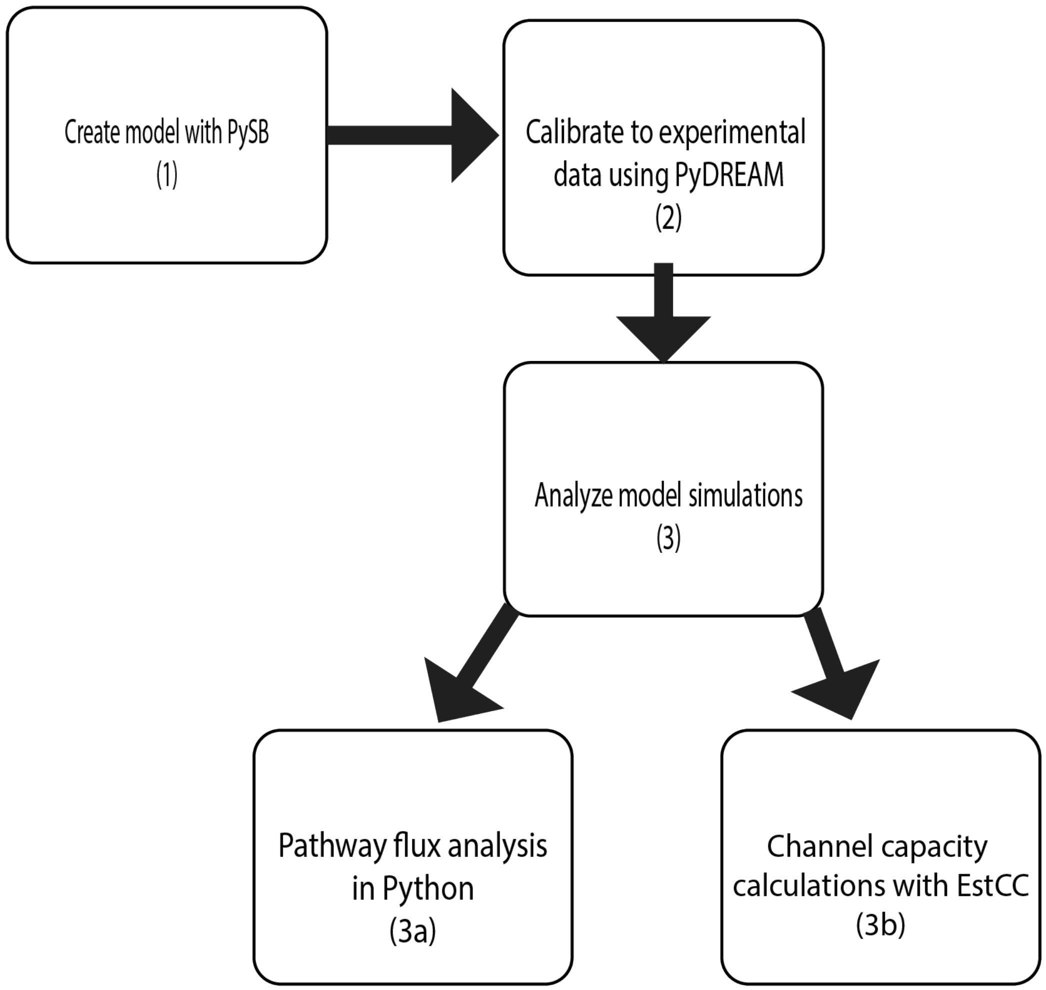

Figure S3: CORM Modeling, Calibration, and Analysis Process. An overview of the entire process of model creation, calibration, and analysis. Model components and relationships between the components are defined as a PySB model. This model is then fit to experimental data using PyDREAM to obtain probability distributions for each fitted parameter. Model simulations corresponding to each parameter set are generated. Pathway flux analysis and channel capacity calculations are then performed on the simulated outputs.

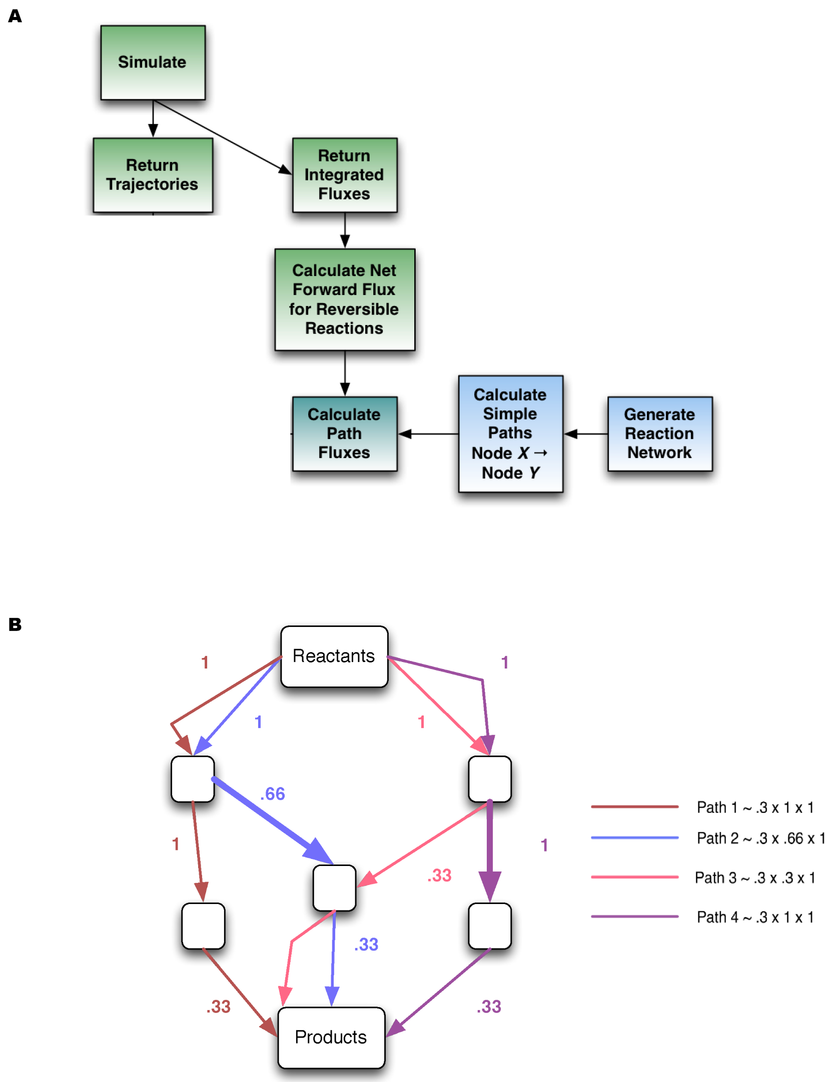

Figure S4: Pathway Flux Analysis. (A) An overview of the process for calculating pathway fluxes from simulations that include reaction fluxes. (B) A more detailed look at calculation of path fluxes for a simple network. Edges are directed towards the flow of the net integrated reaction flux (for bidirectional reactions) or the integrated forward reaction flux (for unidirectional reactions). Edge labels indicate the probability of selecting that reaction path into a node, calculated as the proportion of total flux leaving the node that came from a given reaction. Each color represents a different path.

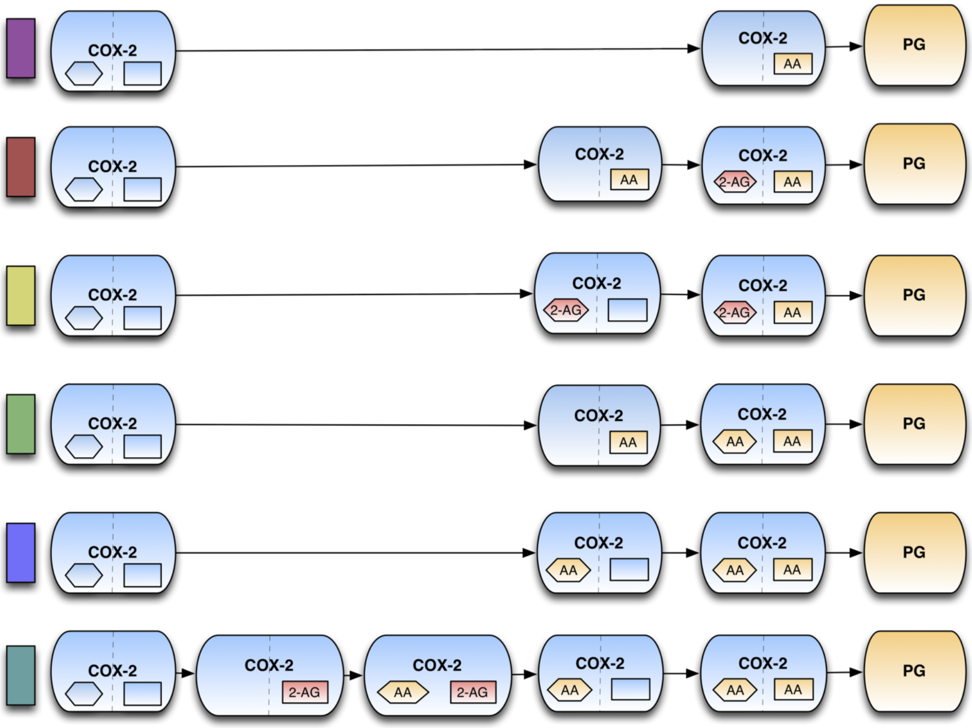

Figure S5: PG Paths. Possible simple (no repeating nodes) paths between COX-2 and PG in CORM. Each path begins with the unbound enzyme and ends with the product PG. Different binding sequences at the allosteric and catalytic sites on the enzyme differentiate the paths.

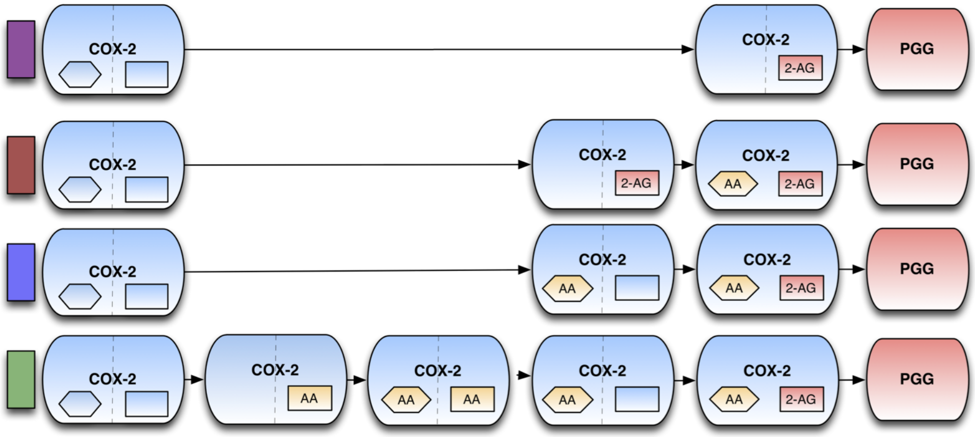

Figure S6: PGG Paths. Possible simple (no repeating nodes) paths from COX-2 to PGG in CORM. Each path begins with the unbound enzyme and ends with the product PGG. Different binding sequences at the allosteric and catalytic sites on the enzyme differentiate the paths.

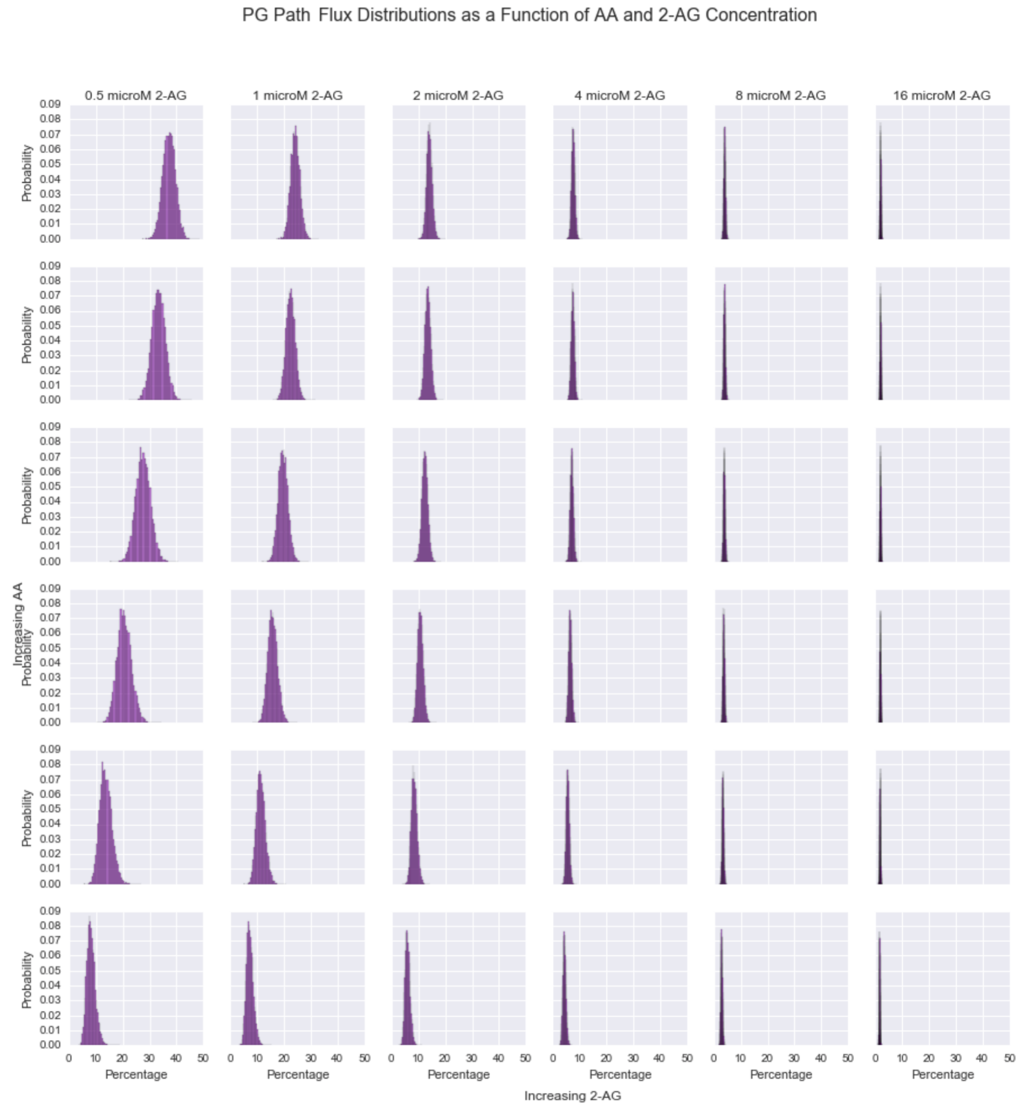

Figure S7: PG Pathway Production Fluxes, Purple Pathway (Fig. S5). Distributions (arising from calibrated parameter uncertainty) of the percentage of the total flux to product PG from the path COX-2 $\leftrightarrow$ COX-2:AA $\rightarrow$ PG at different starting AA and 2-AG concentrations.

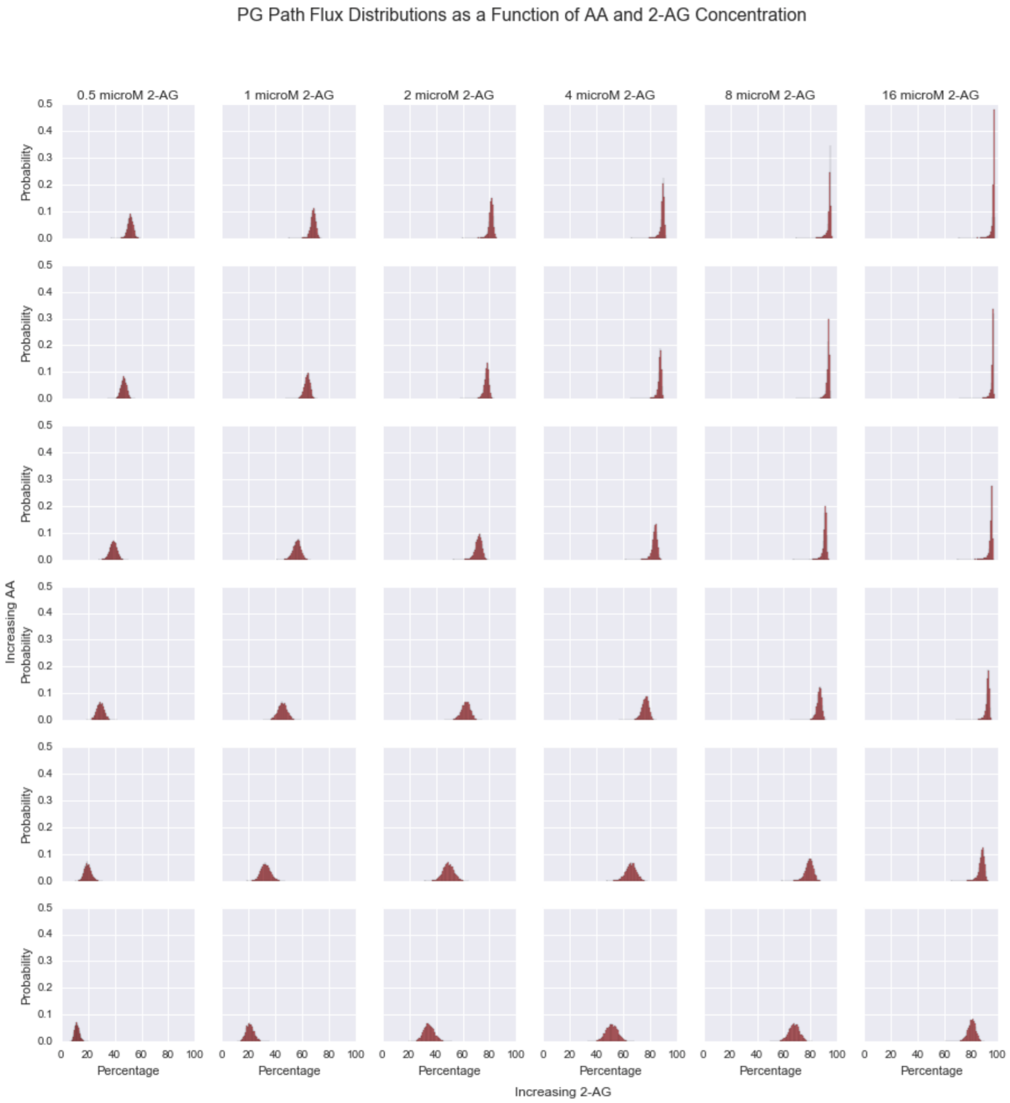

Figure S8: PG Pathway Production Fluxes, Red Pathway (Fig. S5). Distributions (arising from calibrated parameter uncertainty) of the percentage of the total flux to product PG from the path COX-2 $\leftrightarrow$ COX-2:AA $\leftrightarrow$ 2-AG:COX-2:AA $\rightarrow$ PG at different starting AA and 2-AG concentrations.

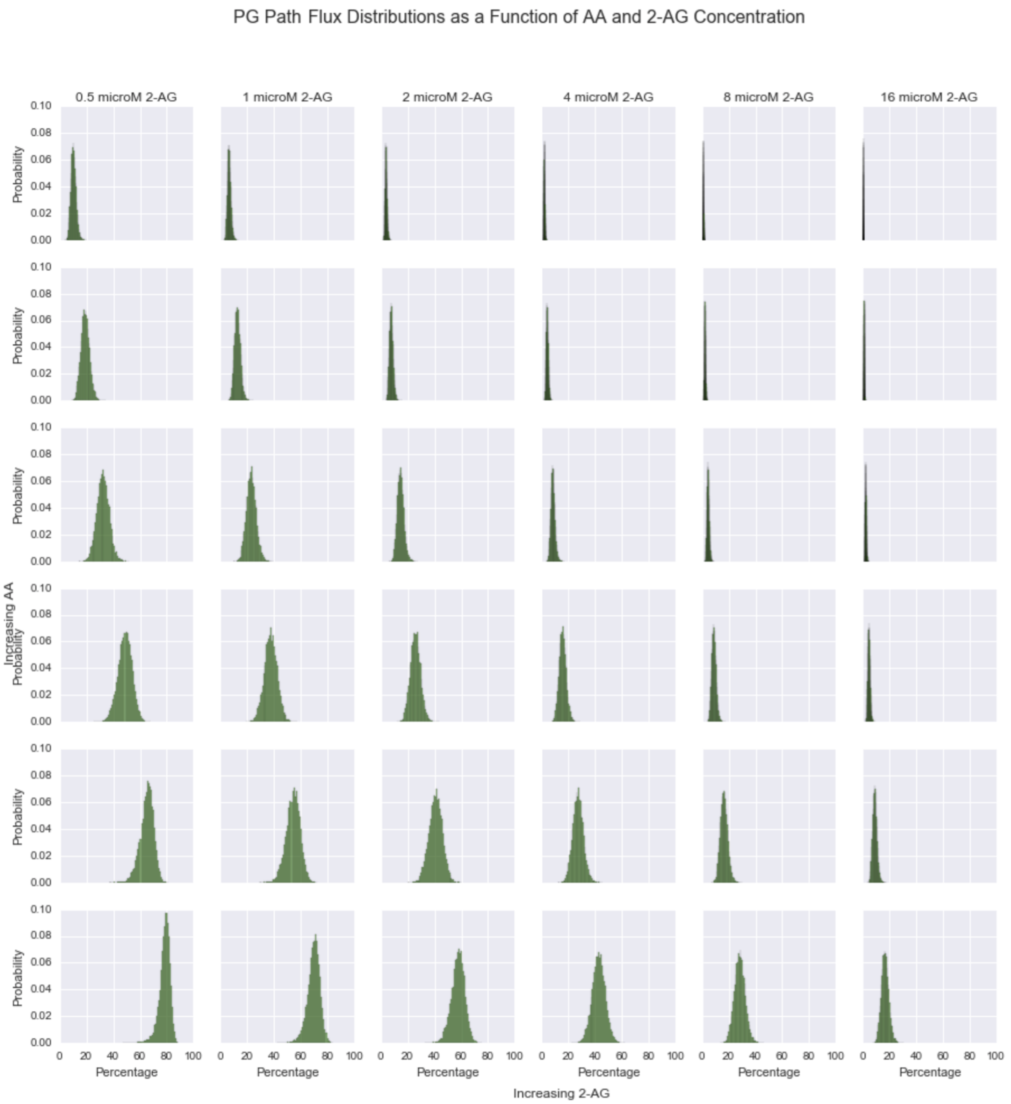

Figure S9: PG Pathway Production Fluxes, Green Pathway (Fig. S5). Distributions (arising from calibrated parameter uncertainty) of the percentage of the total flux to product PG from the path COX-2 ↔ COX-2:AA ↔ AA:COX-2:AA → PG at different starting AA and 2-AG concentrations.

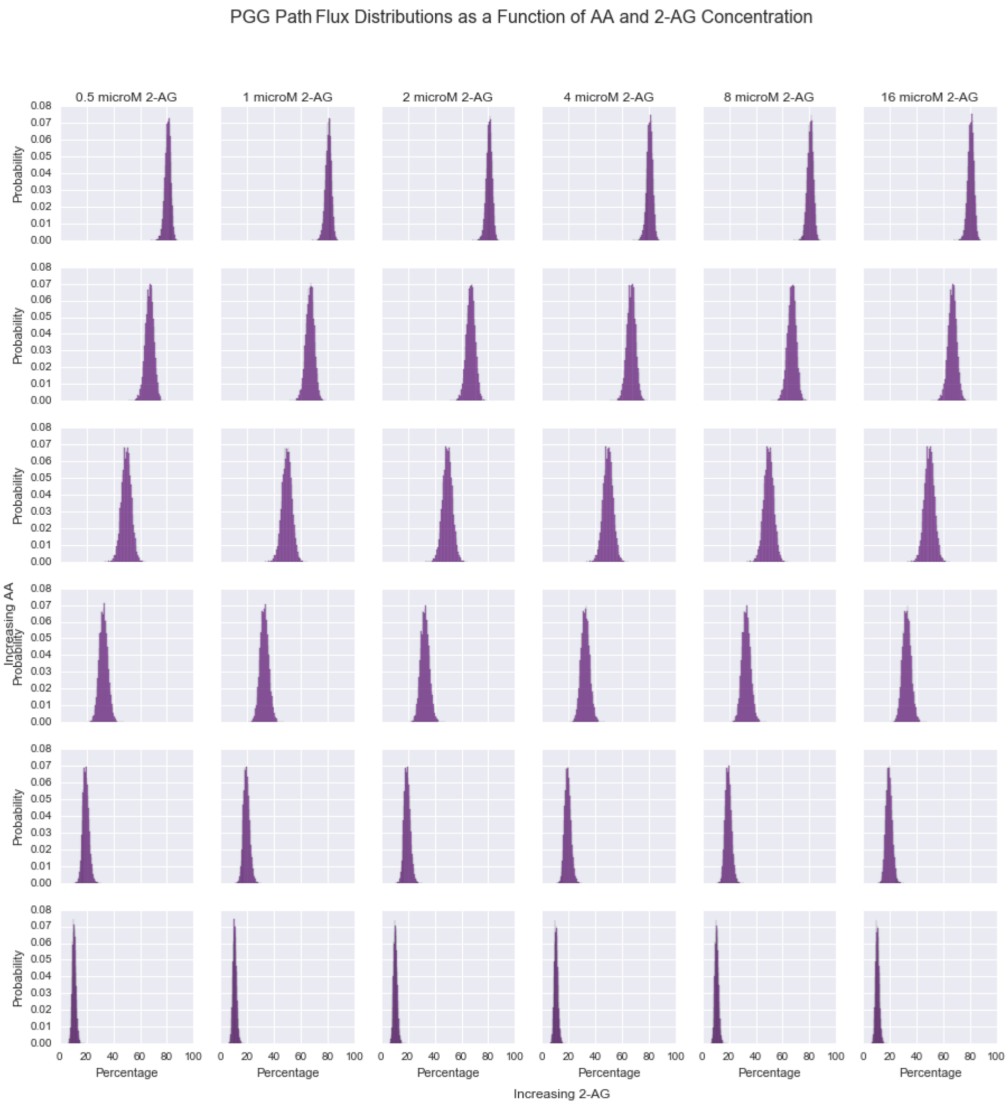

Figure S10: PGG Pathway Production Fluxes, Purple Pathway (Fig. S6). Distributions (arising from calibrated parameter uncertainty) of the percentage of the total flux to product PGG from the path COX-2 $\leftrightarrow$ COX-2:2-AG $\rightarrow$ PGG at different starting AA and 2-AG concentrations.

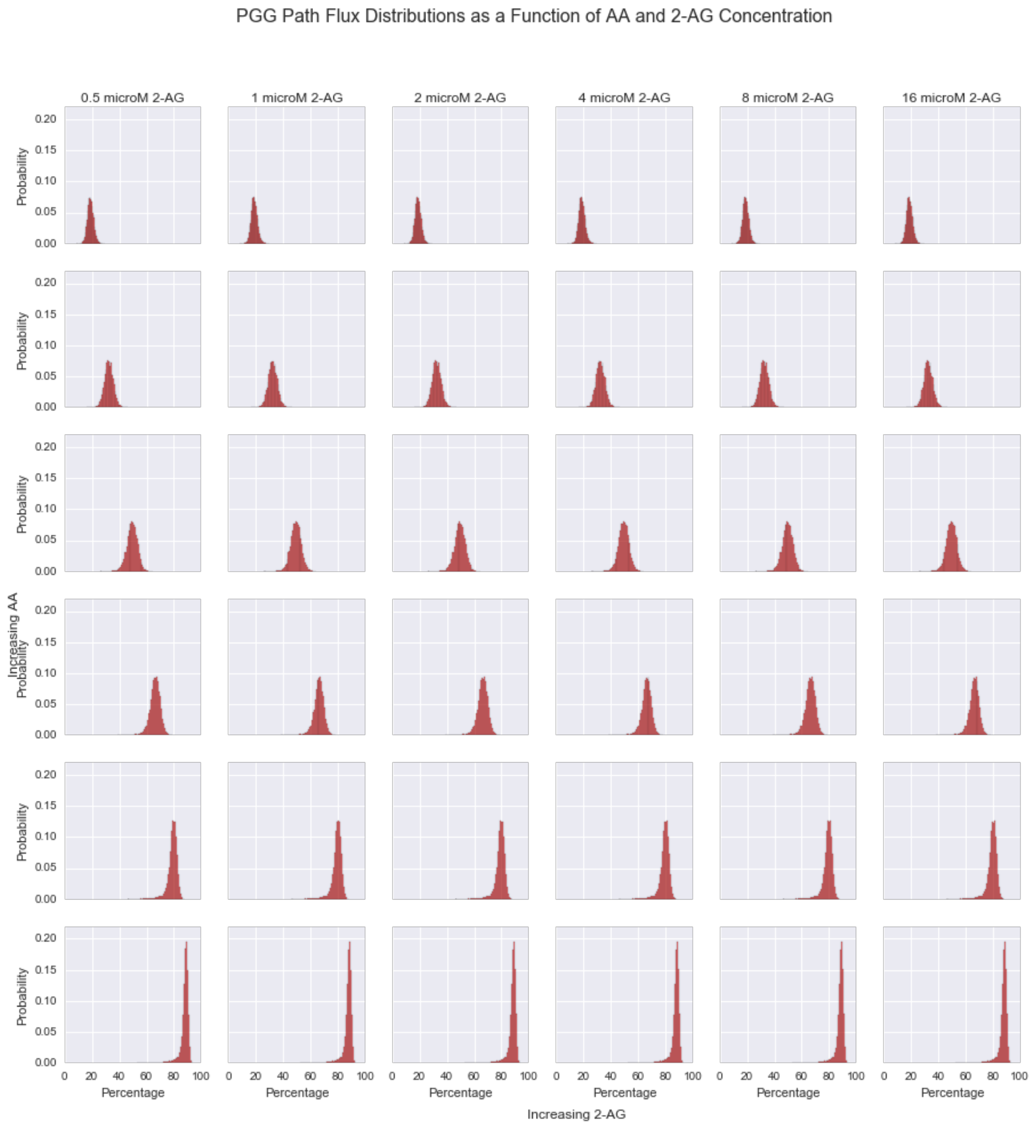

Figure S11: PGG Pathway Production Fluxes, Red Pathway (Fig. S6). Distributions (arising from calibrated parameter uncertainty) of the percentage of the total flux to product PGG from the path COX-2 $\leftrightarrow$ COX-2:2-AG $\leftrightarrow$ AA:COX-2:2-AG $\rightarrow$ PGG at different starting AA and 2-AG concentrations.

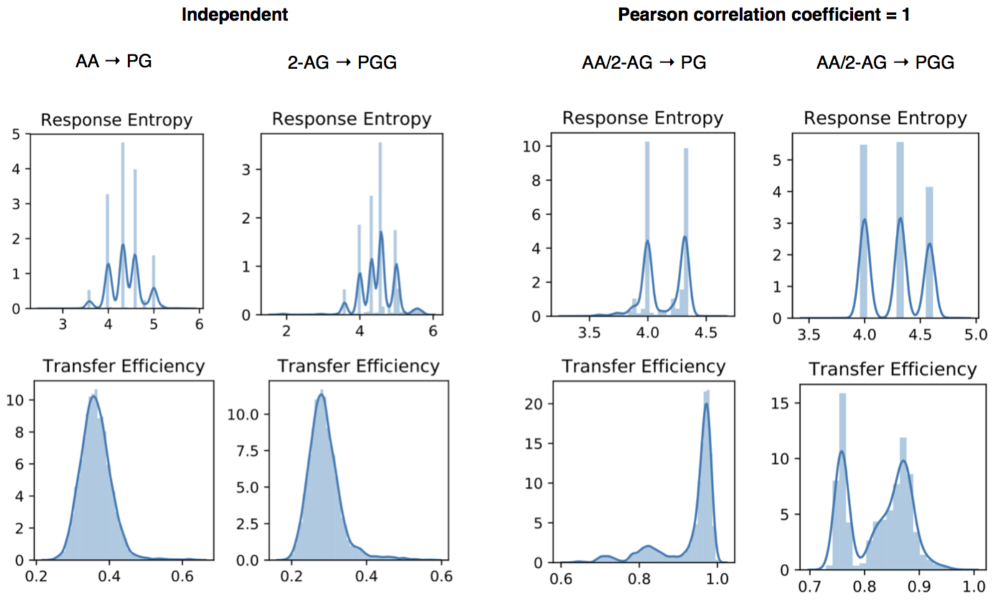

Figure S12: PG and PGG Response Entropy and Transfer Efficiency when AA and 2-AG are Independent or Correlated. Distributions of response entropy under different input correlation and signal/response pairs. While the response entropy spans a similar range with both independent and correlated inputs, the transfer efficiency increases with correlation.

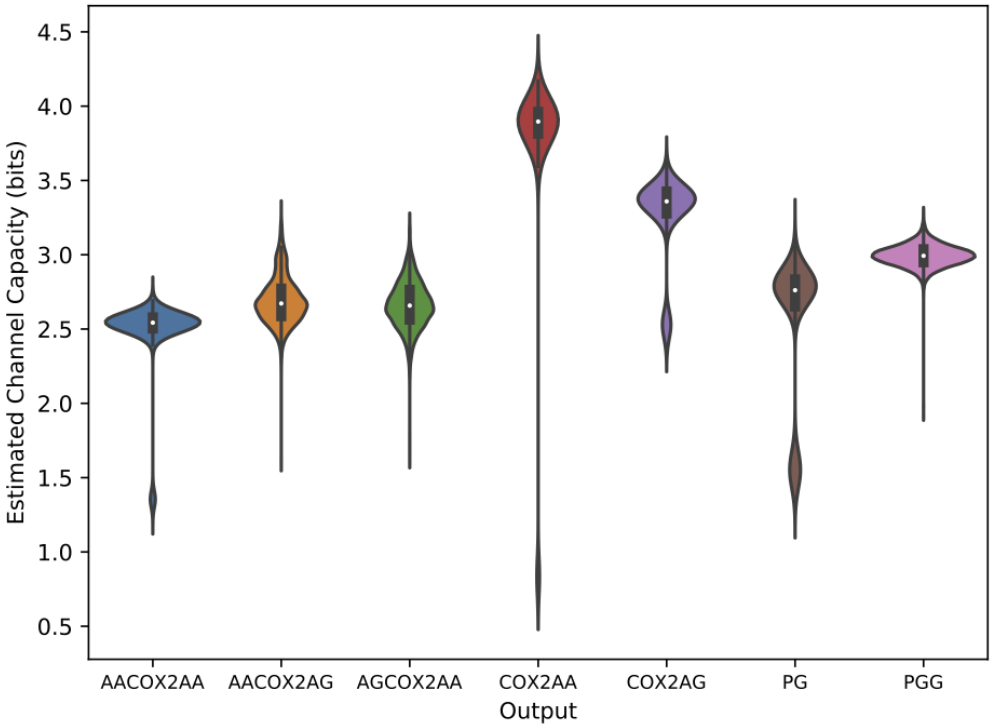

Figure S13: Channel Capacities when Substrate Inputs are Semi-Correlated. Sum of channel capacity from AA and 2-AG to intermediates and final outputs when inputs are semi-correlated (Pearson correlation coefficient = .5). Distributions in channel capacities arise from uncertainty in calibrated parameter values.

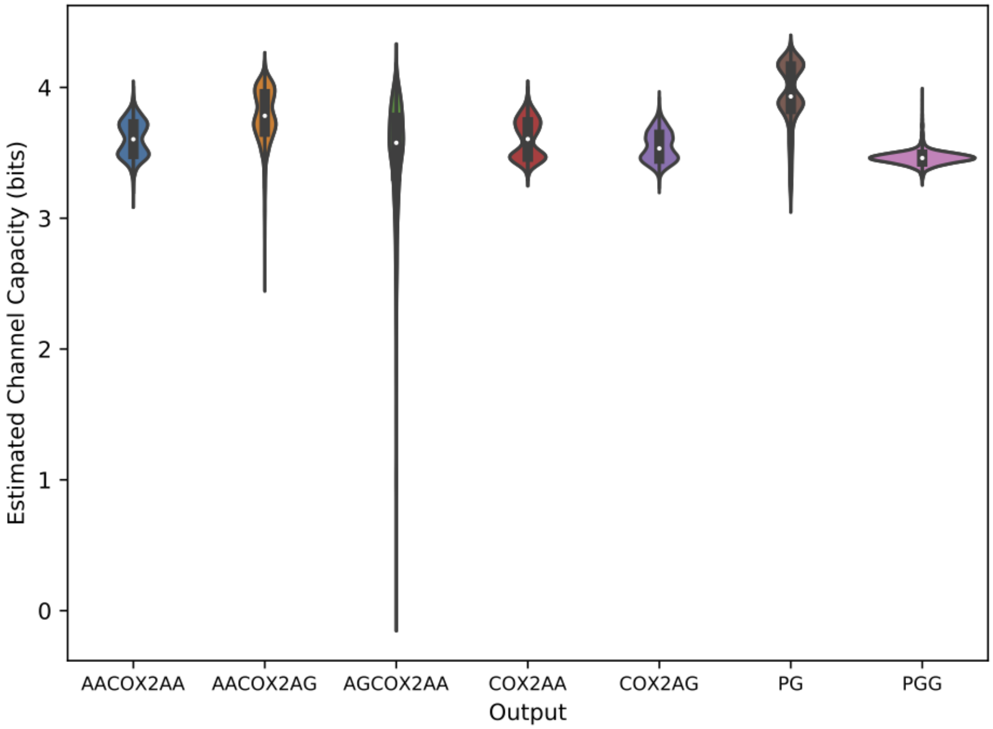

Figure S14: Channel Capacities when Substrate Inputs are Present in a 2:1 AA:2-AG Ratio. Channel capacity from AA and 2-AG to intermediates and final outputs when inputs are present in a 2:1 AA:2-AG ratio. Distributions in channel capacities arise from uncertainty in calibrated parameter values.

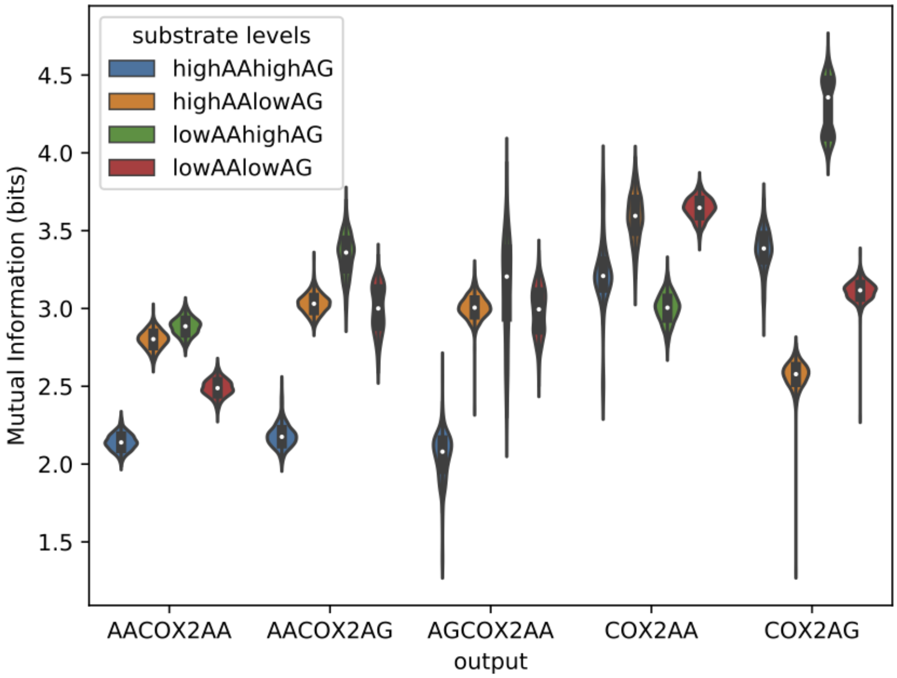

Figure S15: Effect of Substrate Level on Channel Capacities when Inputs are Independent. Sum of channel capacity from AA and 2-AG to intermediates when inputs are varied independently in different regions of substrate space. Distributions in channel capacities arise from uncertainty in calibrated parameter values.

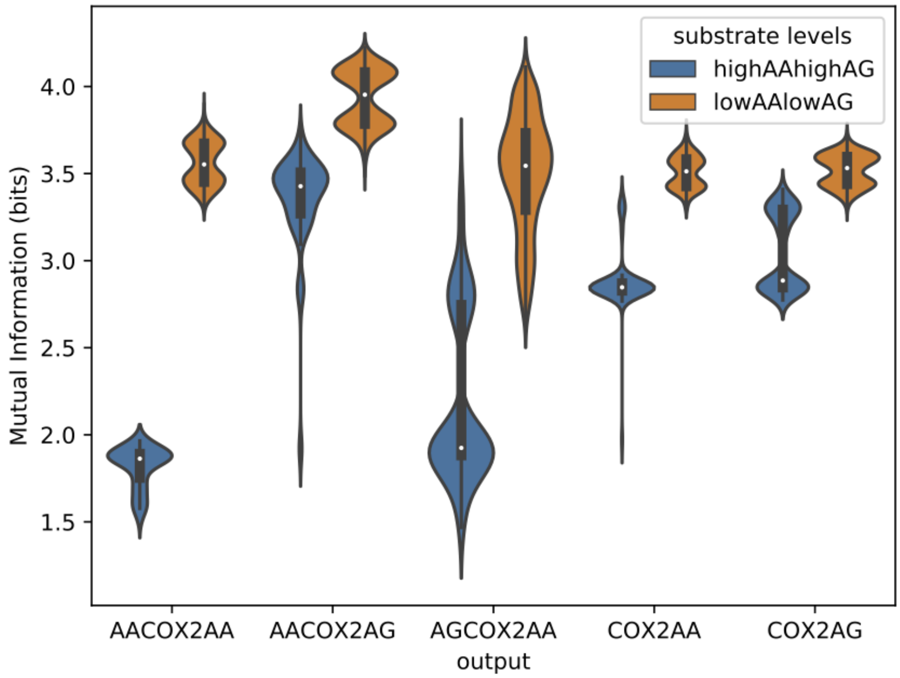

Figure S16: Effect of Substrate Level on Channel Capacities when Inputs are Correlated. Channel capacity from AA and 2-AG to intermediates when inputs are strongly correlated (Pearson correlation coefficient = 1) in different regions of substrate space. Distributions in channel capacities arise from uncertainty in calibrated parameter values.

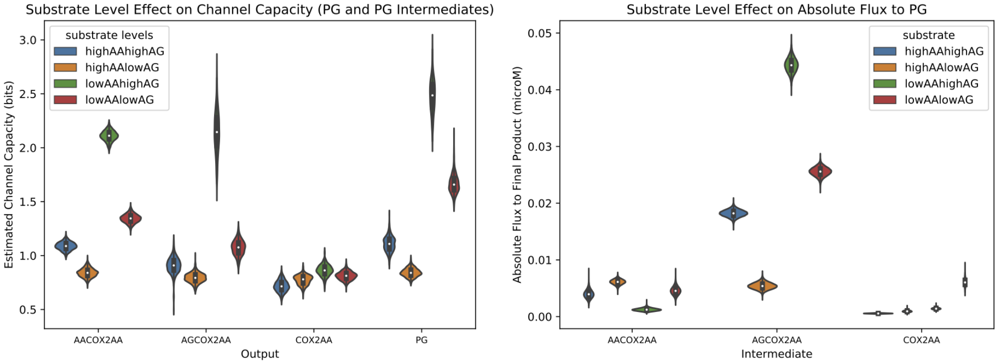

Figure S17: Channel Capacities and Intermediate Fluxes for PG under Different Substrate Levels. Left: channel capacity between independent inputs and PG intermediates at different substrate levels. Right: absolute flux between independent inputs and PG intermediates at different substrate levels.

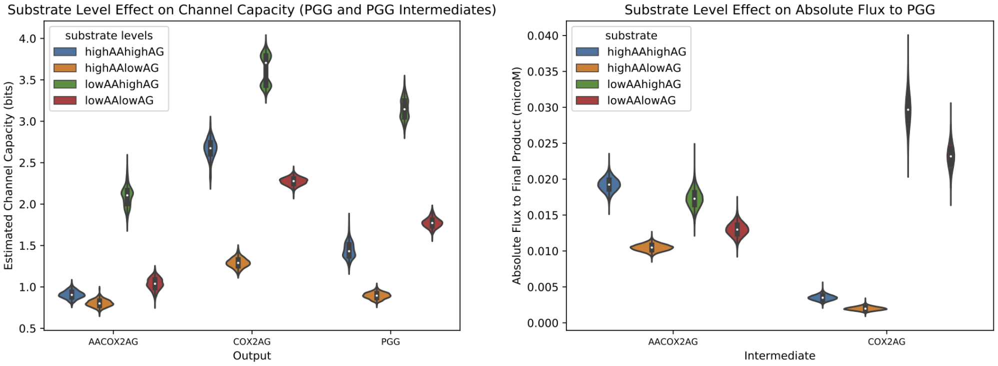

Figure S18: Channel Capacities and Intermediate Fluxes for PGG under Different Substrate Levels. Left: channel capacity between independent inputs and PGG intermediates at different substrate levels. Right: absolute flux between independent inputs and PGG intermediates at different substrate levels.
